# Computational investigation of naturally occurring anticancer agents in regulating Hedgehog pathway proteins

**DOI:** 10.1371/journal.pone.0311307

**Published:** 2024-12-03

**Authors:** Renu Pai, Divijendranatha Reddy Sirigiri, Rajyalakshmi Malempati, Saisha Vinjamuri

**Affiliations:** Department of Biotechnology, BMS College of Engineering, Bengaluru, Karnataka, India; Vignan Pharmacy College, INDIA

## Abstract

Embryonic development in humans is controlled by the Hedgehog pathway, which becomes inactive in mature tissues. Except for tissue maintenance and healing, activation of this pathway results in tumorigenesis with only a few exceptions. The drugs currently in use have shown no effectiveness in blocking the key proteins responsible for tumorigenesis. Therefore, it is crucial to find new inhibitors that can stop the abnormal activation of the pathway. A preliminary Insilco screening of naturally occurring compounds was carried out to identify potential inhibitors of the pathway. Docking of seventeen naturally occurring antitumorigenic compounds against the four key proteins of the regulatory proteins of the Hedgehog pathway using AutoDock v4.2.6 software was carried out. Liriodenine exhibited the strongest binding affinity towards three out of the four regulatory proteins (-7.61 kcal/mol with Smoothened, -8.14 kcal/mol with Patched-I, and -6.15 kcal/mol with Gli-II) of the Hedgehog pathway, whereas 2’,4-dihydroxy-3-methoxychalcone displayed the highest binding affinity of -7.04 kcal/mol with the Sonic Hedgehog protein. Additional molecular dynamic simulation was conducted using Gromacs with Liriodenine and 2’,4-dihydroxy-3-methoxy chalcone. Every protein-ligand complex underwent simulation using v5.1.4 software for a duration of 100 nanoseconds. The findings from the simulation indicate that Liriodenine and 2’,4-dihydroxy-3-methoxy chalcone form a strong bond with their corresponding protein. Our findings show that the two aforementioned molecules have potential as new inhibitors of the pathway and should be further investigated in both invitro and in vivo experiments.

## 1. Introduction

The hedgehog pathway is one of the conserved pathways leading to cell differentiation during embryogenesis [1, 2] after which it only gets activated, mostly in the skin, during cell repair [3]. The research on tumorigenesis has shown that it shares its mechanism of action with embryogenesis starting with proliferation, differentiation and migration [4]. Thus the anomalous activation of the embryogenic pathway such as the Hedgehog pathway leads to tumorigenesis in the adult tissue [5] causing cancer in the lungs, breasts, brain, skin, etc [6–10]. The research on inhibition of this pathway is directed at the four regulatory proteins namely the Smoothened (Smo), the Patched I (Ptch I) receptor, the Sonic hedgehog (SHH) and the glioma-associated oncogenes II (Gli II) protein [11–14].

Many small molecule inhibitors have been identified for each of the four proteins. These inhibitors consist of both natural and synthetic compounds [15]. Some of the antagonists approved for clinical use are cyclopamine, vismodegib, taladegib, sonidegib, saridegib, glasdegib, TAK-441, and BMS 833923 [16]. Although cyclopamine was not easily absorbed through oral administration and had subpar pharmacokinetics, other compounds were found to be ineffective because of drug resistance resulting from mutations in the Smoothened and Gli proteins. Previous research has investigated the Smoothened protein’s interactions with various drug candidates such as Exelixis® BMS 833923 (XL 139) [17], 2’,4’,5’,3,4- pentamethoxychalcone [18], hydroxychloroquine [19] and 9-Hexyl -2- (4- (pyridin-4-yl) piperazin-1-yl)- 6- (4- (trifluoromethoxy) phenyl)- 9H- purine (4s) [20]. The Patched protein has also been examined for its susceptibility to compounds like 20(S)-Ginsenoside Rg3 [21] and its sterol sensing domain [22] through docking studies. Natural compounds such as Zerumbone, Curcumin, and Butein [23] have been aimed at the Sonic hedgehog protein and discovered to possess a binding affinity, showing potential for oral cancer therapy [23]. Compounds like GANT-61 [24] and 3-(3,4-bis((4-chlorobenzyl)oxy)phenyl)-5,7-dimethoxy-4H-chromen-4-one [25] have demonstrated potential as antagonists against the Gli protein in inhibiting the growth of medulloblastoma. Drug candidates such as epigenetic modulators [26] and natural alkaloids [27] have also been analyzed in silico to potentially target multiple proteins within this pathway.

It is crucial to emphasize that most drug leads exclusively focus on the Smoothened protein. Therefore, the approach is to focus on various proteins within the pathway and, at the same time, combat resistance using naturally occurring phytochemicals.

Chalcones are the polyphenols produced in plants as the precursors of flavonoids in the flavonoid biosynthetic pathway. Their therapeutic potential includes antibacterial, antimalarial, anti-leishmanial, antiviral and also its anticancer activity [28–32]. A simple condensation reaction of aryl ketones with aromatic aldehydes or substituting one of the many displaceable hydrogens can result in new chalcone derivatives [33]. Glabrescione B, an isoflavone derivative was testified as Smo antagonist and Gli inhibitor from a library of natural compounds [34]. Additionally, NL-103, a hybrid substance created by merging pharmacophores from GDC-0449 and a histone deacetylase inhibitor, demonstrated the ability to inhibit the movement of Smo to the primary cilium [35]. Chalcones have been recently combined with anti-cancer pharmacophores to assess their effectiveness in combating drug resistance. The hybrids created with azoles such as imidazole, thiazole, pyrazole, and tetrazole have demonstrated enhanced specificity and improved effectiveness in addressing drug resistance [36–39].

2, 4’-Dihydroxy chalcone and 2’,4-dihydroxy-3-methoxychalcone are among the chalcones that have been extensively researched for their anticancer properties. These compounds were obtained from *Polyalthia cauliflora* (Annonaceae) and have shown promising effects against breast cancer cell lines [40]. The researchers investigated the ability of the methanolic leaf extract of *Polyalthia longifolia* to induce apoptosis in HeLa cells [41]. The extract was found to upregulate pro-apoptotic proteins and downregulate anti-apoptotic proteins, leading to HeLa cell growth inhibition. Fissistin, Isofissistin, and Pedicin, which are chalcones extracted from *Fissistigma* of the *Annonaceae* family, have been shown to be successful in combating human epithelial carcinoma cells [42]. Licochalcone A and trans-chalcone derived from *Glycyrrhiza* (*Fabaceae*) exhibit notable anti-cancer effects on the breast cancer cell line MCF-7 [43]. Xanthohumal isolated from *Humulus lupulus* L., has exhibited tremendous anticancer potential against gastric cancer by inducing apoptosis and inhibiting NF-κB signaling [44]. The research on Ginkgetin from *Ginkgo biloba* shows that it improves the DDP (Cisplatin) action by inducing ferroptosis in nonsmall cell lung cancer (NSCLC) [45]. Even though enough literature explains how chalcones work as anti-cancer agents, their influence on the hedgehog pathway, which is responsible for starting tumor formation, has not been well recorded.

The present research focuses seventeen specific naturally-occurring compounds, primarily chalcones, with known anti-cancer properties as reported in previous studies. The chalcones chosen act as regulators of pathways like NF-κB, PI3K/Akt, ABCG2, RSK2, and JNK/c-jun in different types of cancer cells [46–48]. We utilize computational methods to examine how they can influence the Hedgehog pathway’s four regulatory proteins. Molecular docking is conducted with chosen compounds and the four pathway proteins, followed by molecular dynamic simulations of the protein-ligand complex with the strongest binding affinity.

We find that chalcones show good binding strength with all four proteins in the pathway, creating hydrogen bonds and showing a steady interaction. ADMET analysis was conducted concurrently to highlight the drug-likeness of the compounds.

## 2. Materials and methods

### 2.1. Selection of the ligand compounds and retrieving the structures

Selection of ligand compounds: From the literature review of secondary metabolites with anticancer properties (S1 Table), we obtained the structures of seventeen chalcone molecules and the standard cyclopamine from the PubChem database [49] as detailed in Table 1. The ligands were transformed into pdb format from sdf structures with the help of the web-based Openbabel tool (S2 Table).

**Table 1:** List of ligands and their details.

Sl.No.	Name	PubChem ID	Molecular Formula	Source plant	Family
1	2’, 4’-dihydroxy chalcone	5376979	C_15_H_12_O	*Polyalthia cauliflora* [40]	*Annonaceae*
2	2’,4-dihydroxy-3-methoxy chalcone	5973690	C_16_H_14_O_4_	*Polyalthia cauliflora* [40]	*Annonaceae*
3.	Cyclohexanone, 2,3,3-trimethyl-2-(3-methyl-1,3-butadienyl)-, (Z)-	5371302	C_14_H_22_O	*Polyalthia cauliflora* [40]	*Annonaceae*
4.	Liriodenine	10144	C_17_H_9_NO_3_	*Polyalthia cauliflora* [50]	*Annonaceae*
5.	Fissistin	9981988	C_28_H_34_O_6_	*Fissistigma launoginosum* [42]	*Annonaceae*
6.	Isofissistin	10027544	C_28_H_34_O_6_	*Fissistigma launoginosum* [42]	*Annonaceae*
7.	Pedicin	5901370	C_18_H_18_O_6_	*Fissistigma launoginosum* [42]	*Annonaceae*
8.	flavokawain A	5355469	C_18_H_18_O_5_	*Piper methysticum* [51]	*Piperaceae*
9.	flavokawain B	5356121	C_17_H_16_O_4_	*Piper methysticum* [51]	*Piperaceae*
10.	flavokawain C	6293081	C_17_H_16_O_5_	*Piper methysticum* [51]	*Piperaceae*
11.	Hydroxyderricin	6438503	C_21_H_22_O	*Angelika keiskei* [52]	*Umbelliferae*
12.	Isobavachalcone	5281255	C_20_H_20_O_4_	*Angelika keiskei* [53]	*Umbelliferae*
13.	Licochalcone A	5318998	C_21_H_22_O_4_	*Angelika keiskei* [54]	*Umbelliferae*
14.	Xanthoangelol	643007	C_25_H_28_O_4_	*Angelika keiskei* [55]	*Umbelliferae*
15.	Xanthoangelol B	10409180	C_25_H_28_O_5_	*Angelika keiskei* [56]	*Umbelliferae*
16.	Xanthoangelol F	6479088	C_26_H_30_O_4_	*Angelika keiskei* [53]	*Umbelliferae*
17.	1-Pthalanone	6885	C_8_H_6_O_2_	*Lawsonia inermis* [57]	*Lythraceae*
18.	Cyclopamine	442972	C_27_H_41_NO_2_	*Veratrum californicum* [58]	*Liliaceae*

Table with name, PubChem, molecular formula, source, family of selected lead molecules and cyclopamine with their respective PubChem ID. The structures of each of the chalcone and the standard cyclopamine is given in S2 Table.

Selection of protein: The PDB structures of the proteins, the human Smoothened protein (PDB id: 4JKV), the patched I protein (PDB id: 6D4H), the sonic hedgehog protein (PDB id: 6D4J) and Gli II protein (PDB id: 2GLI) were retrieved from RCSB Protein Data Bank (RCSB PDB) [59]. The molecular docking tool AutoDock 4.2.6 suite was used in the docking analysis [60]. All four proteins were prepared for the docking analysis by removal of co-crystallized water molecules along with the cofactors. The Gasteiger charges of all the proteins were calculated and hydrogen atoms were added using AutoDock Tools. Hydrogen atoms were added to the target protein for correct ionization and tautomeric states of amino acid residues and the nonpolar hydrogens were then merged. The Gasteiger charge was assigned to the ligand [61]. The modified structures obtained were converted to PDBQT format in ADT for AutoDock calculations.

### 2.2. Docking of the ligands

For docking, the Lamarckian Genetic Algorithm was implemented with a population size of 25 docking and 2.5 million energy evaluations [62]. All other parameters were run with default settings such as crossover rate and mutation rate. The grid size for specifying the search space was prepared with the dimension of 126 × 126 × 126 centered on the macromolecule with a default grid point spacing of 0.375 Å [63]. Precisely calculated grid maps were obtained using AutoGrid, which stores a grid of interaction energy based on the interaction of the ligand atom probes with the receptor target. The similarity conformations of docked structures were measured by computing root mean square deviations between the coordinates of the atoms and creating clustering of the conformations based on the RMSD values. The lowest binding energy conformation in all clusters was considered the most favorable docking pose and the molecular interactions of the chalcones with the proteins were visualized using the AutoDock Tools [64].

### 2.3. Molecular simulation studies

Post docking, molecular simulations were carried out. The Gromacs v5.1.4 package [65] was used to apply the CHARMM36 force field [66] to generate the topology and molecular structure files of the protein and the ligand molecules. The binding complex of the protein and the ligand molecule that contributed the least binding energy was obtained using AutoDock 4.2 and was simulated in a neutral environment by adding an appropriate number of sodium or chlorine counter ions. A dodecahedron box was created with a 1.0 Å distance between the protein surface and the box boundary. Then the box was solvated with the TIP3P water model under coulomb type, PME (Particle Mesh Ewald electrostatics). For fixing all bond lengths in the system the Linear Constraint algorithm has been used by using the all-bonds option under constraints (important to use this option when dt > 0.001ps). The system was equilibrated beginning with the protein atom restrained simulations having 100ps equilibration dynamics of the solvent molecules at 300K. The entire system was equilibrated at room temperature for 1000 ps [67]. The equilibration was performed with a periodic boundary condition in the NPT ensemble at 5 K to 298 K with Berendsen temperature coupling and constant pressure 1 Å with isotropic molecule-based scaling [68]. A time step of 1ps and a non-bond interaction cutoff radius of 1 Å were considered. Finally, the production MD run is set for 100 ns and the data generated is analyzed. The interactions were visualized using Discovery Studio 16.

### 2.4. ADMET analysis

The SMILES format of each of the selected compounds along with cyclopamine was obtained from the Pubchem database and converted into a dataset. The dataset was used as an input to acquire the molecular ADMET properties and bioactivity score of the compounds using StarDrop software and SWISS ADME online tool [69].

## 3. Results

### 3.1. Binding studies of the compounds using molecular docking

The 17 selected chalcones were docked with four proteins of the Hedgehog pathway viz., Patched I, Smoothened, Gli II and Sonic Hedgehog, using AutoDock 4.2 software and their binding affinities were compared with standard cyclopamine (Table 2). The molecular interactions present between the ligand and the protein were visualized and a 2D diagram of the interaction was plotted.

**Table 2:** Binding energy score of the chalcones with the respective proteins.

Sl. No.	Chalcone	Patched I kcal/mol	Smoothened kcal/mol	Gli II kcal/mol	Sonic Hedgehog kcal/mol
1	2’,4’-dihydroxychalcone	-6.46	-6.61	-4.94	-6.82
2	**2’,4-dihydroxy-3-methoxy chalcone**	-6.59	-6.4	-5.77	**-7.04**
3	Cyclohexanone, 2,3,3-trimethyl-2-(3-methyl-1,3-butadienyl)-, (Z)-	-6.29	-6.35	-5.93	-6.14
4	Fissistin	-4.54	-5.34	-4.74	-5.03
5	Flavokawain A	-5.58	-6.39	-4.68	-5.45
6	Flavokawain B	-5.71	-6.28	-4.63	-5.1
7	Flavokawain C	-6.15	-5.97	-4.2	-6.34
8	Hydroxyderricin	-6.4	-6.27	-5.24	-6.33
9	Isobavachalcone	-7.53	-6.49	-4.67	-6.32
10	Isofissistin	-4.4	-5.42	-4.53	-4.8
11	Licochalcone A	-6.49	-5.9	-6.12	-5.43
12	**Liriodenine**	**-8.14**	**-7.61**	**-6.15**	-6.92
13	1-Phthalanone	-4.51	-5.11	-4.27	-4.93
14	Pedicin	-4.79	-5.43	-4.62	-4.31
15	Xanthoangelol	-6.57	-5.76	-4.81	-6.36
16	Xanthoangelol B	-6.3	-6.04	-4.49	-4.52
17	Xanthoangelol F	-5.66	-5.6	-4.47	-4.78
18	Cyclopamine	-9.45	-9.43	-8.01	-7.68

The binding affinity of the ligand that shows the highest binding affinity is highlighted in bold. 2’,4-dihydroxy-3-methoxy chalcone shows the highest binding affinity with Sonic Hedgehog protein while Liriodenine shows the highest binding affinity with the other three proteins (Smoothened, Patched I and Gli II).

It is evident in the table above that Liriodenine demonstrates the highest effectiveness against Hedgehog pathway proteins. It demonstrates the lowest energy usage for three out of the four proteins examined. It interacts with the Patched I protein with a binding energy of -8.14 kcal/mol, whereas it binds with the Smoothened and the Gli II protein with binding energies of -7.61 kcal/mol and -6.15 kcal/mol, respectively.

The 2’,4-dihydroxy-3-methoxy chalcone had a notable impact on the Sonic hedgehog protein by expending -7.04 kcal/mol of binding energy. In this case, Liriodenine displayed strong binding affinity, although it was slightly lower than 2’,4-dihydroxy-3-methoxy chalcone, which had a binding energy of -6.92 kcal/mol.

#### 3.1.1. Docking of Liriodenine with Smoothened protein, Patched I and Gli II proteins

**Fig 1:**
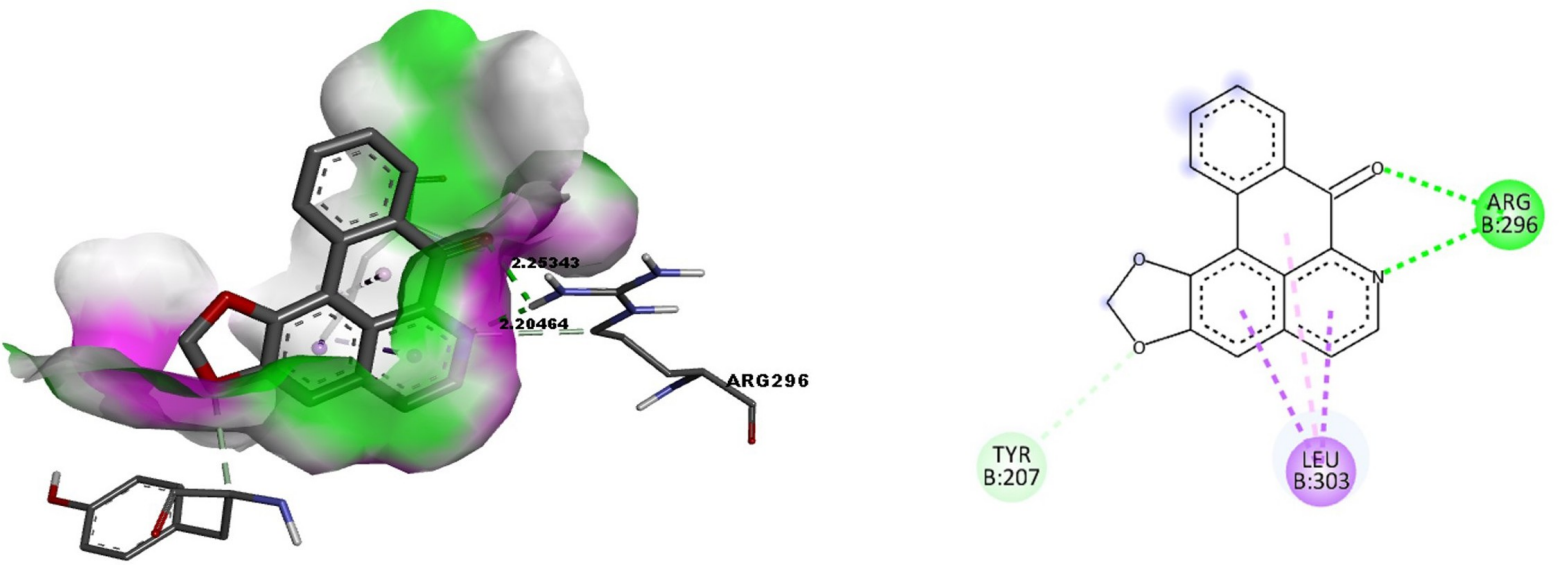
Docking of Liriodenine with the Smoothened protein. Fig 1A. The binding pocket surface amino acid Arginine296 of the Smoothened protein forms the hydrogen bonds with the ligand Liriodenine. Fig 1B. 2D representation of the interaction between amino acids of the Smoothened protein and the ligand Liriodenine.

The binding of Liriodenine with the Smoothened protein involved two hydrogen bonds shared with a single active site residue ARG296 of lengths 2.25 and 2.205 Å (Fig 1A and 1B).

**Fig 2:**
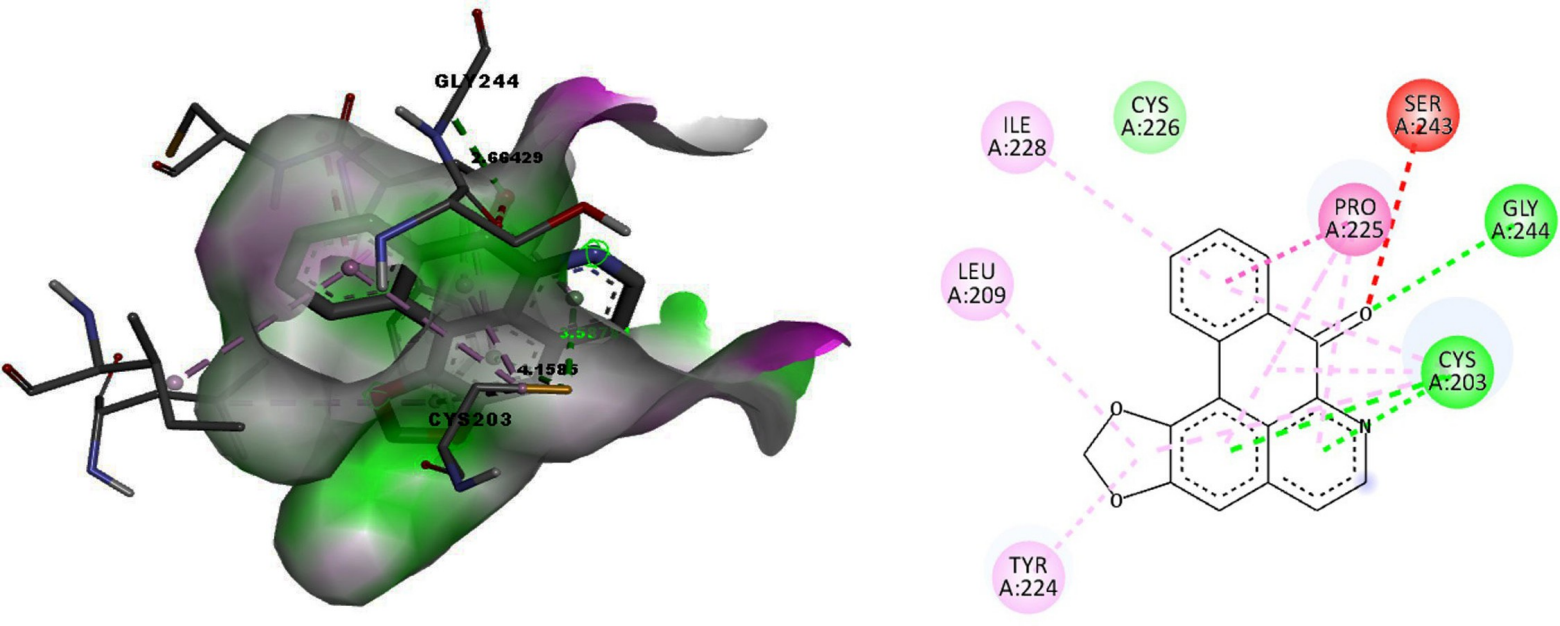
Docking of Liriodenine with the Patched I protein. Fig 2A. The binding pocket surface amino acid Leucine 777 of the Patched protein forms the hydrogen bond with the ligand Liriodenine. Fig 2B. 2D representation of the interaction between amino acids of the Patched protein and the ligand Liriodenine.

Liriodenine formed hydrogen bonds with the active site residues CYS203 and GLY244 of Patched I protein, with bond lengths of 3.58 and 2.65 Å, respectively (Fig 2A and 2B). During each of the 25 runs, there was at least one hydrogen interaction observed between the ligand and the protein during docking.

**Fig 3:**
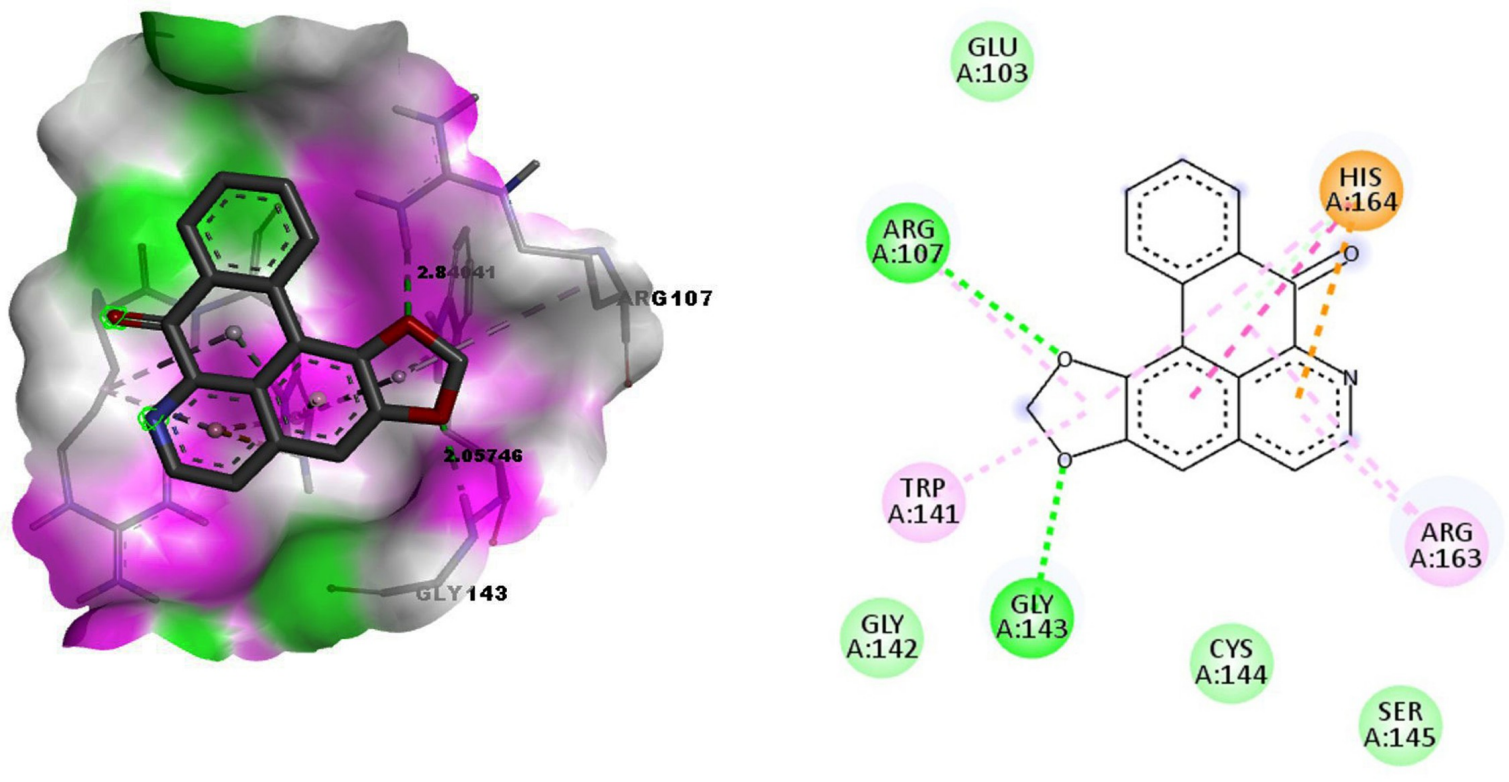
Docking of Liriodenine with the Gli II protein. Fig 3A. The binding pocket surface amino acids Glycine 143 and Arginine 107 of the Gli II protein form the hydrogen bond with the ligand Liriodenine. Fig 3B. 2D representation of the interaction between amino acids of the Gli II protein and the ligand Liriodenine.

With Gli II protein the binding of Liriodenine was observed at active site residue GLY143 and Arg107 through hydrogen bonding with the bond length of 2.057 and 2.84 Å respectively (Fig 3A and 3B).

#### 3.1.2. Docking of 2’,4-dihydroxy-3-methoxy chalcone with Sonic Hedgehog protein

**Fig 4:**
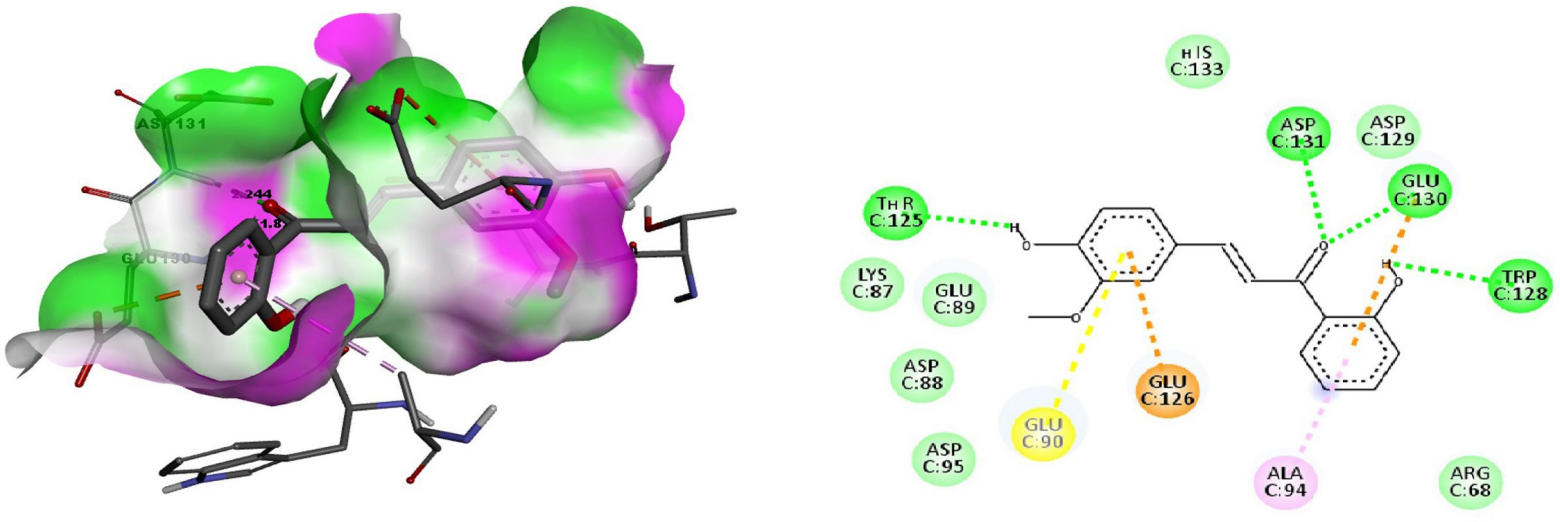
Docking of 2’,4-dihydroxy-3-methoxy chalcone with the Sonic Hedgehog protein. Fig 4A. The binding pocket surface amino acid Threonine 125, Tryptophan 128, Glutamate 130 and Aspartate 131 of the Sonic Hedgehog protein form the hydrogen bond with the ligand 2’,4-dihydroxy-3-methoxy chalcone. Fig 4B. 2D representation of the interaction between amino acids of the Sonic Hedgehog protein and the ligand Liriodenine.

2’,4-dihydroxy-3-methoxy chalcone emerged as the compound with the highest binding affinity to the Sonic Hedgehog protein with a binding energy of -7.04 kcal/mol being the lowest among all tested compounds. It was discovered that it forms connections with active site residue ASP131 and GLU130 of SHH protein via two hydrogen bonds, with bond lengths of 2.244 and 1.827Å each (Fig 4A and 4B).

On the flip side, the protein had a binding energy of -6.92 kcal/mol with Liriodenine. Pedicin had the highest amount of binding energy at -4.31 kcal/mol among all the compounds.

While cyclopamine had high affinities for all four proteins, it only formed hydrogen bonds with Gli II protein. It created two hydrogen bonds, with bond distances of 2.035 and 1.86 Å, with ASN227 and ASN222 residues, respectively.

### 3.2. Molecular dynamic simulations for interaction analysis

Due to its strong binding affinity with Smoothened protein, Patched I protein, and Gli II protein, Liriodenine was chosen for additional molecular dynamics simulations. Similarly, 2’,4-dihydroxy-3-methoxy chalcone, which had the highest binding affinity with sonic Hedgehog protein, was also selected for further studies.

#### 3.2.1. Molecular simulation of Liriodenine with Smoothened protein

**Fig 5:**
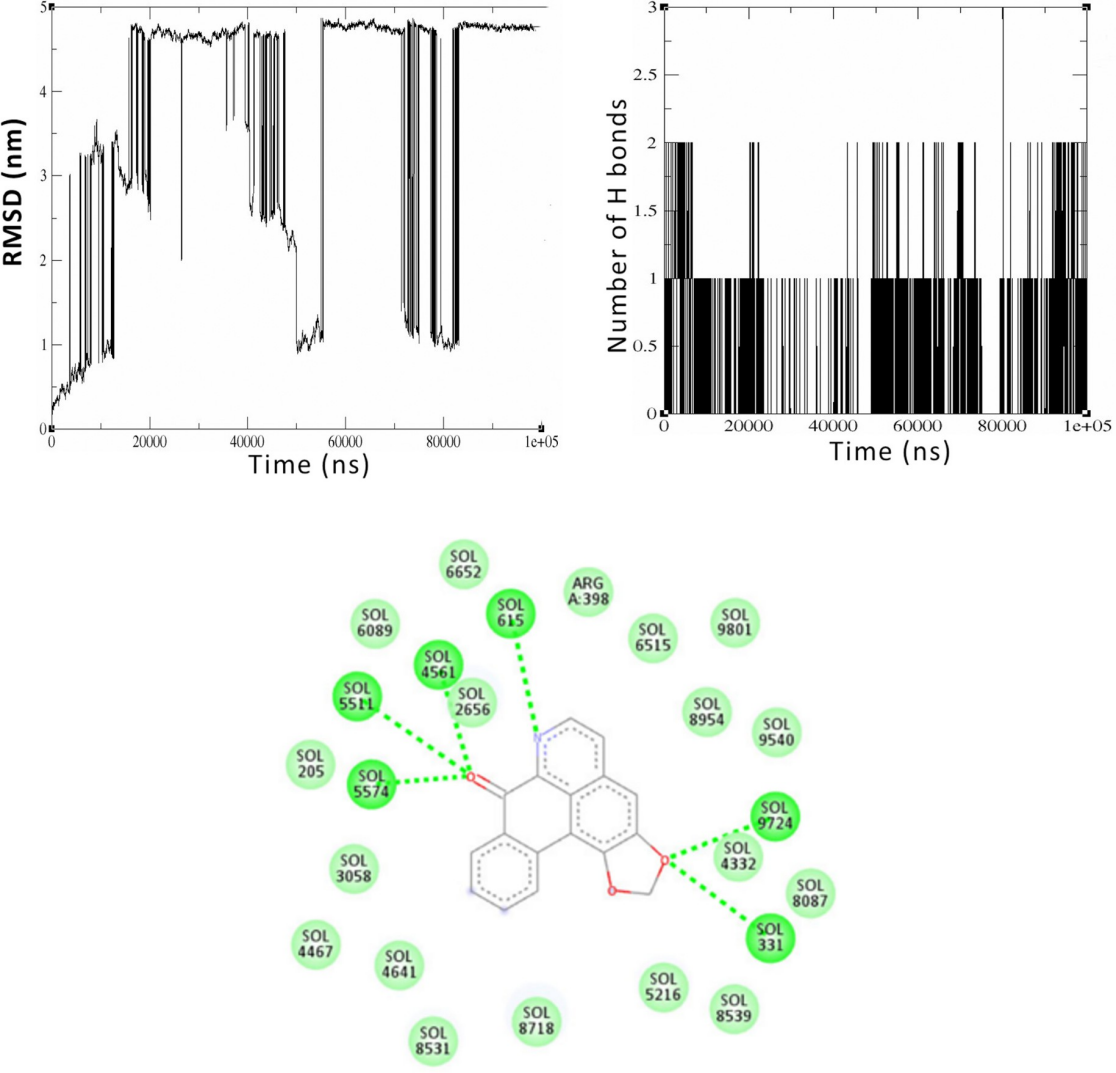
Simulation of interaction between of Liriodenine with the Smoothened protein. Fig 5A. RMSD graph representing the structural deviation of the backbone structure of the Smoothened protein after 100 ns simulation with Liriodenine, Fig 5B. Hydrogen bond analysis plot derived from the trajectory analysis of 100 ns simulation between the Smoothened protein and Liriodenine, Fig 5C. The 2D representation after 100 ns shows the bonds formed between the chalcone Liriodenine and the surrounding solvent molecules.

The stability of the interaction between the Smoothened protein and Liriodenine was shown in a 100 ns simulation. The RMSD (Root Mean Square Deviation) measures how much the protein’s structure changed from its initial form to the one after interacting with Liriodenine. The Smoothened protein shows the greatest and most erratic RMSD difference compared to the other four proteins (Fig 5A). It starts at a small 0.2 nm then jumps to 3 nm and further to 4.8 nm by 18 ns. This cycle of fluctuations persists until it eventually reaches a steady point at 82 nanoseconds. The findings indicate that the interaction affects the protein’s backbone structure, indicating a shift in conformation. The number of hydrogen bonds formed between biomolecules remains relatively constant during the 100 ns simulation, with most forming 1–2 hydrogen bonds between them (Fig 5B) The simulation’s trajectory analysis reveals that the protein establishes three hydrogen bonds with the compound Liriodenine at approximately 80 ns. The compound’s interaction with surrounding solvent molecules is depicted in the 2D representation (Fig 5C) after the 100 ns simulation.

#### 3.2.2. Molecular simulation of Liriodenine with Patched I protein

**Fig 6:**
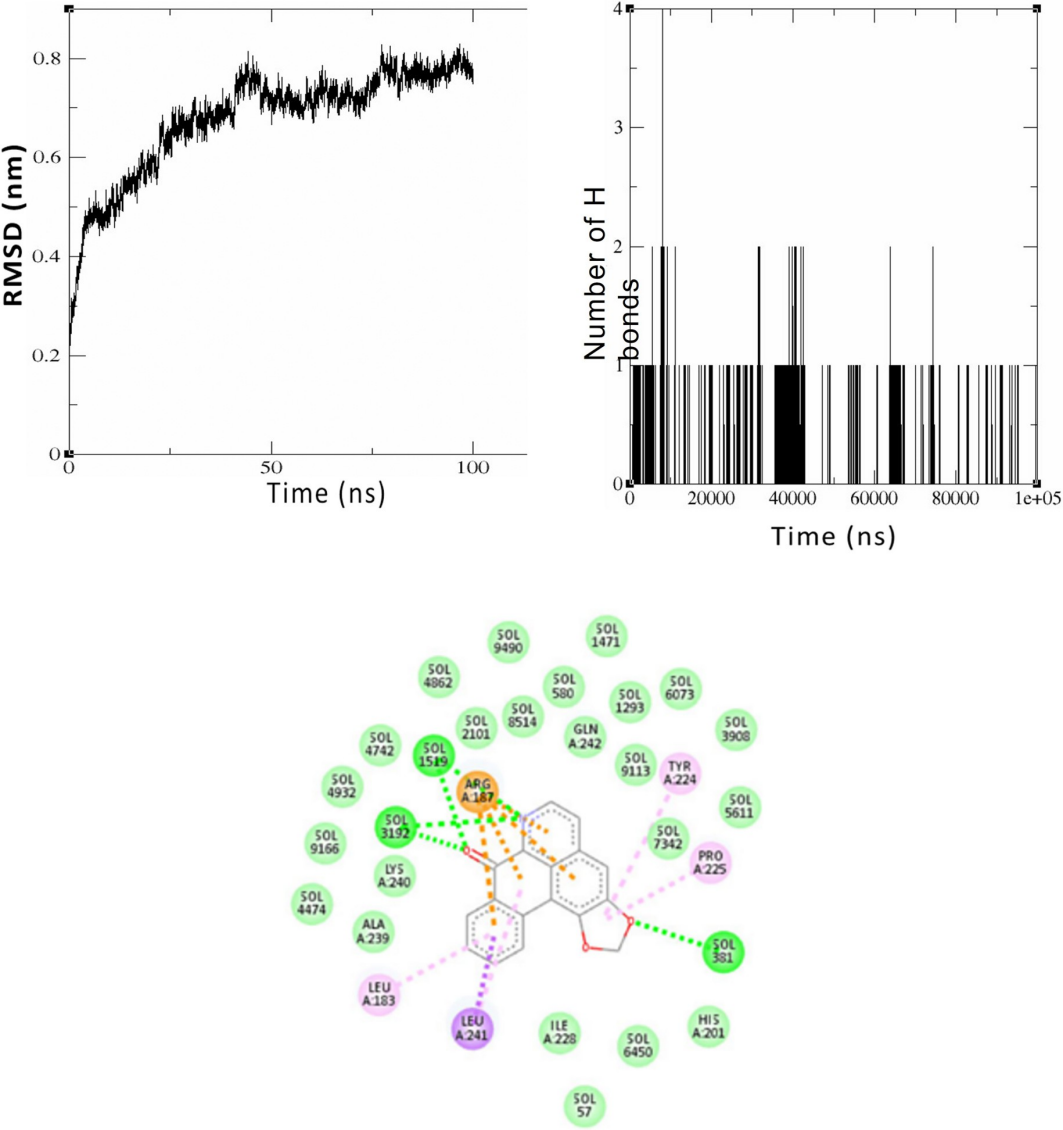
Simulation of interaction between of Liriodenine with the Patched I protein. Fig 6A. RMSD graph representing the structural deviation of the backbone structure of the Patched I protein after 100 ns simulation with Liriodenine, Fig 6B. Hydrogen bond plot derived from the trajectory analysis of 100 ns simulation between Patched I protein and Liriodenine, Fig 6C. The 2D representation of the bonds formed between the A chain of the Patched I protein and the chalcone Liriodenine after 100 ns.

In the initial 10 nanoseconds, there is a stronger association observed between Liriodenine and the Patched I protein. The protein exhibits the smallest variation in the backbone structure compared to all other proteins in the pathway. During the initial 10 nanoseconds, it rotates with a minimum of 0.5 nanometers. Despite the growing difference, it progresses steadily and slowly until it reaches 0.8 nm at 100 ns, as shown in Fig 6A.

The protein exhibits consistent hydrogen bonding interaction lasting 45 ns, which diminishes gradually by 100 ns as shown in Fig 6B. It can create up to four hydrogen bonds with the compound within 8 nanoseconds. The protein and Liriodenine continue interacting for over 100 ns. The hydrophobic association of Leucine 183, Arginine 187, Tyrosine 224, Proline 225, and Leucine 241 with the Patched I protein is depicted in the 2D representation in Fig 6C.

#### 3.2.3. Molecular simulation of Liriodenine with Gli II protein

**Fig 7:**
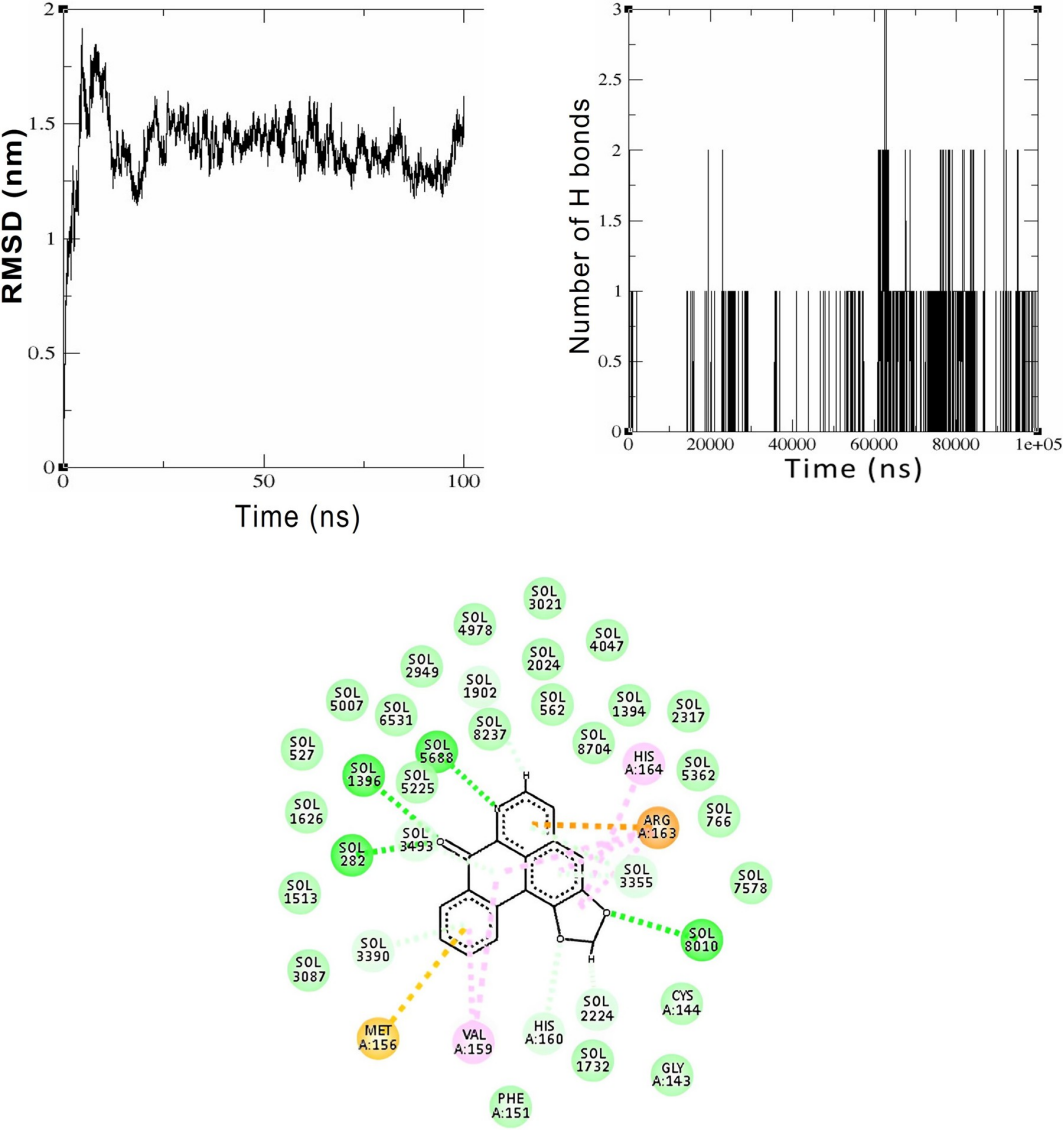
Simulation of interaction between of Liriodenine with the Gli II protein. Fig 7A. RMSD graph representing the structural deviation of the backbone structure of the Gli II protein after 100 ns simulation with Liriodenine, Fig 7B. Hydrogen bond plot derived from the trajectory analysis of 100 ns simulation between Gli II protein and Liriodenine, Fig 7C. The 2D representation of the bonds formed between the amino acids of the Gli II protein and the chalcone Liriodenine after 100 ns.

The protein Gli II, which is the smallest of all proteins, consistently interacts with the compound Liriodenine. In the first 10 ns of the 100 ns simulation, the protein deviates up to approximately 1.8 nm, as depicted in Fig 7A, on the RMSD plot. Afterward, the protein is observed to have a strong bond with the compound for the entire simulation. A steady hydrogen bonding typically forms following 20 ns of interaction. Analysis of the trajectory over 100 ns shows the creation of a minimum of 1–2 hydrogen bonds between the pair. The protein forms three hydrogen bonds with Liriodenine at approximately 63 ns and 92 ns, as illustrated in Fig 7B. Even after 100 nanoseconds, the amino acids Methionine 156, Valine 159, Arginine 163, and Histidine 164 maintain hydrophobic interaction with the protein, as illustrated in Fig 7C.

#### 3.2.4. Molecular simulation of 2’,4-dihydroxy-3-methoxy chalcone with Sonic Hedgehog protein

**Fig 8:**
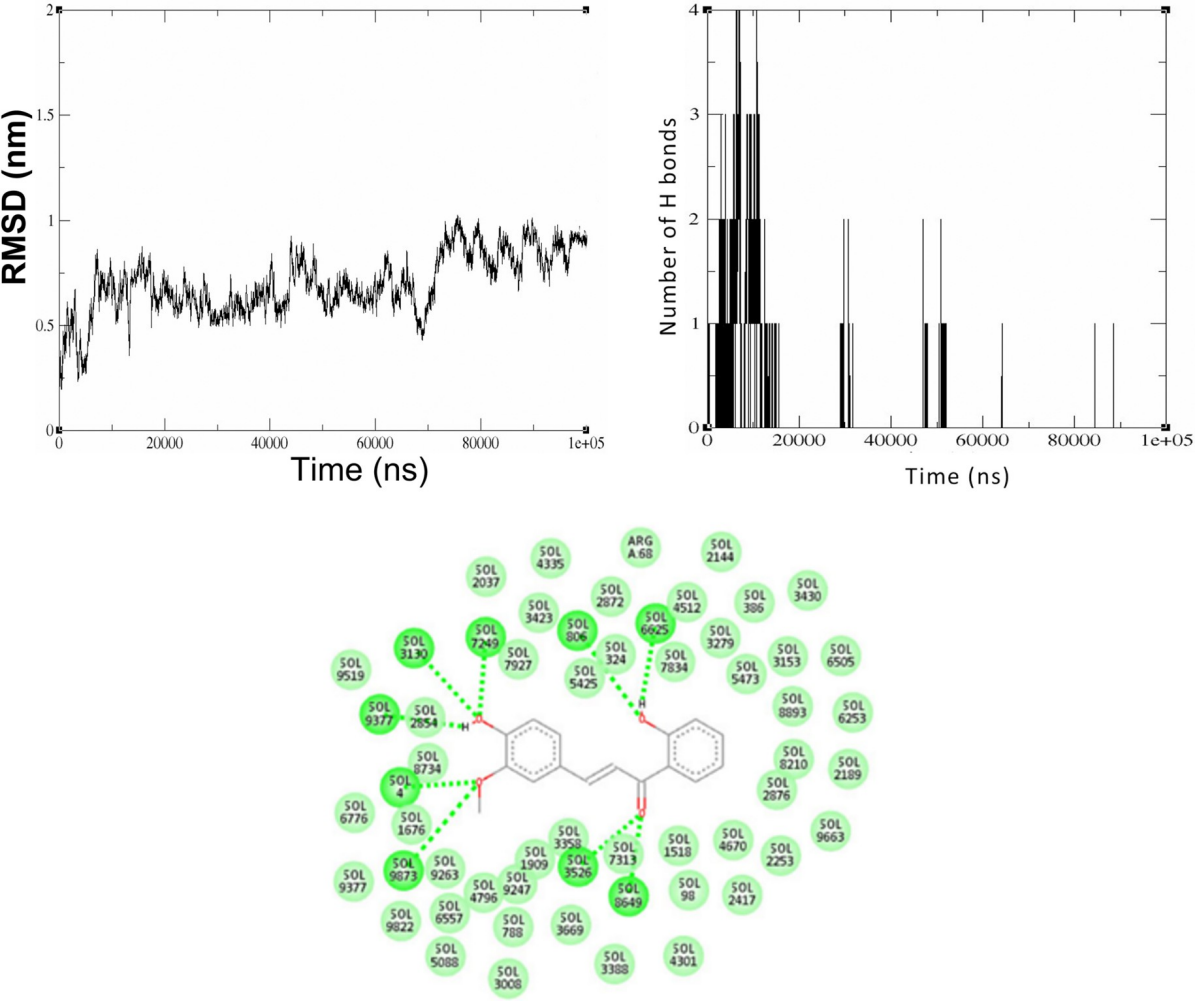
Simulation of interaction between of 2’,4-dihydroxy-3-methoxy chalcone with the Sonic Hedgehog protein. Fig 8A. RMSD graph representing the structural deviation of the backbone structure of the Sonic Hedgehog protein after 100 ns simulation with 2’,4-dihydroxy-3-methoxy chalcone, Fig 8B. Hydrogen bond analysis plot derived from the trajectory analysis of 100 ns simulation between the Sonic Hedgehog protein and 2’,4-dihydroxy-3-methoxy chalcone, Fig 8C. The 2D representation after 100 ns shows the bonds formed between the chalcone and the surrounding solvent molecules.

The Sonic Hedgehog protein binds with the least binding energy to the compound 2’,4-dihydroxy-3-methoxy chalcone. The graph of RMSD for the Sonic Hedgehog protein shows that the interaction remains stable with a deviation of 0.5nm for the initial 10 ns. During the simulation, there is a deviation of up to 1 nm, followed by a decrease back to 0.9 nm by the end of 100 ns, as shown in Fig 8A.

The RMSD, reflecting alterations in the protein’s backbone, indicates that binding of 2’,4-dihydroxy-3-methoxy chalcone to the protein does not impede its structural integrity. The process starts by the Sonic Hedgehog protein forming hydrogen bonds with Aspartate 131, Glutamate 130, and Threonine 135 when interacting with 2’,4-dihydroxy-3-methoxy chalcone. The analysis plot shows clearly that the protein forms four hydrogen bonds with the compound at 6ns and at approximately 12 ns. The number of hydrogen bonds formed between the molecules diminishes over time, and by 100 ns there are no hydrogen bonds present as shown in Fig 8B. After 100 ns, the compound no longer interacts with the Sonic Hedgehog protein. Observations from Fig 8C show that only solvent molecules bond with the protein.

Table 3 displays a summary of the interactions of the four proteins and the two chalcones, as assessed using docking and simulation studies. These computational techniques enable a thorough examination of how well ligands bind to proteins and their specificity, which is useful in the process of discovering and developing drugs. The table shows the binding energy parameter, which indicates how strong the protein interacts with the compound. An attractive interaction between two molecules is suggested by a negative binding energy, whereas a repulsive interaction is indicated by a positive binding energy. The amount of hydrogen bonds between the protein and the compound is crucial, as hydrogen bonding plays a vital role in establishing the stability of protein-ligand complexes. The Smoothened and Patched I proteins have interaction energies of -102.8 kcal/mol and -129.3 kcal/mol, respectively, with Liriodenine, while the interaction energy with the Gli II protein is -96.7 kcal/mol. The 2’,4-dihydroxy-3-methoxy chalcone had an interaction energy of -18.7 kcal/mol with the Sonic Hedgehog protein. The findings indicate that the connection between Liriodenine and the specific proteins, primarily Patched I, demonstrates increased strength due to the number of hydrogen bonds established. During the 100 ns simulation, there are four hydrogen bonds established between 2’,4-dihydroxy-3-methoxy chalcone and the Sonic Hedgehog protein. However, it is noted that there is minimal interaction after 18 nanoseconds due to its decreased intermolecular interaction energy.

**Table 3:** Docking and simulation results.

Protein	Chalcone	Binding energy (kcal/mol)	Number of H-Bonds	Estimated inhibition constant (*Ki*) nM	Total intermolecular interaction energy kcal/mol
Smoothened	Liriodenine	-7.61	3	2.62	-102.8859
Patched I	Liriodenine	-8.14	4	1.07	-129.3926
Gli II	Liriodenine	-6.15	3	3.23	-96.7363
Sonic Hedgehog	2’,4-dihydroxy-3-methoxy chalcone	-7.04	4	6.95	-18.72618

The results of the docking and simulations summarized as table shows that the naturally occurring compounds interact with proteins of the Hedgehog pathway with good interaction energy. The highest interaction was observed between Liriodenine and Patched I at -129.39 kcal/mol.

### 3.3. ADMET analysis

Table 4 presents the findings of the virtual screening conducted on seventeen chalcone molecules and cyclopamine for drug-like characteristics using the Swiss ADME tool and StarDrop.

**Table 4:** ADMET and physicochemical properties of the selected chalcones.

Sl. No.	Name	Log P	TPSA	MW	n HA	n HBD	n rotb	Log S	GI a	BBB p	SA
1	2’,4’-dihydroxy chalcone	2.91	60.68	242.27	3	3	3	-3.85	High	Yes	2.49
2	2’,4-dihydroxy-3-methoxy chalcone	3.37	66.76	270.3	4	2	4	-3.9	High	Yes	2.61
3	Cyclohexanone, 2,3,3-trimethyl-2-(3-methyl-1,3-butadienyl)-, (Z)-	2.78	17.07	206.32	1	0	2	-3.66	High	Yes	3.94
4	Fissistin	7.61	85.23	466.57	6	2	9	-6.18	High	No	4.7
5	flavokawain A	3.83	65	314.34	5	1	6	-4.17	High	Yes	2.87
6	flavokawain B	3.77	55.77	284.31	4	1	5	-4.11	High	Yes	2.73
7	flavokawain C	3.29	76	300.31	5	2	5	-3.96	High	Yes	2.77
8	Hydroxyderricin	4.88	66.76	338.4	4	2	6	-5.32	High	Yes	3.1
9	Isobavachalcone	4.81	77.75	324.38	4	3	5	-5.1	High	No	3.03
10	Isofissistin	6.66	85.23	466.57	6	2	9	-6.18	High	No	4.7
11	licochalcone A	4.85	66.76	338.4	4	2	6	-4.98	High	Yes	3.23
12	Liriodenine	3.3	48.43	275.26	4	0	0	-4.25	High	No	2.77
13	1-Phthalanone	1.5	26.3	134.13	2	0	0	-1.62	High	Yes	1.34
14	Pedicin	3.25	85.23	330.34	6	2	6	-4.03	High	No	3.04
15	Xanthoangelol	6.66	77.75	392.5	4	3	8	-6.44	High	No	3.57
16	Xanthoangelol B	5.29	97.98	408.49	5	4	9	-5.84	High	No	4.03
17	Xanthoangelol F	6.73	66.76	406.52	4	2	9	-6.66	High	No	3.63
18	Cyclopamine	3.39	41.49	411.6	3	2	0	-4.61	High	Yes	6.3

According to Table 4, it is evident that the 17 chosen compounds possess a molecular weight of under 500 g/mol and a favorable Topological Polar Surface Area (TPSA) of less than 140 Å, suggesting a high level of Gastro-Intestinal (GI) absorptivity. The molecules all possess the required amount of hydrogen donors (5) and acceptors (10) needed for their bioavailability. These properties are essential for the passive diffusion of molecules through the membrane during absorption and distribution. All candidate molecules had fewer than 10 rotatable bonds, indicating their flexibility and facilitating their absorption by the system. It has been demonstrated that the permeation rate and Log P value of drug candidates are controlled by the TPSA value and number of rotatable bonds.

The Log P value, which represents lipophilicity, plays a crucial role in determining the absorption of potential drug candidates. Out of the seventeen molecules that were tested, thirteen had a Log P index value below 5 which is considered acceptable, whereas five had higher values. The findings indicate that Liriodenine and 2’,4-dihydroxy-3-methoxy chalcone have Log P values of 3.3 and 3.37, respectively, which are comparable.

The bioavailability of a compound greatly depends on its solubility in water. Four molecules have Log S values ranging from -2 to -4, indicating solubility, while nine have values between -4 and -6, showing moderate solubility. There were only four molecules with a Log S value greater than -6 and up to -10, indicating they have low solubility. Liriodenine and 2’,4-dihydroxy-3-methoxy chalcone exhibited a solubility index of -4.25 and -3.9, respectively, suggesting favorable bioavailability. Eight compounds did not show blood-brain barrier permeation (BBB p), while the others tested positive for it. The findings indicate that Liriodenine does not pass through the BBB, while 2’,4-dihydroxy-3-methoxy chalcone does show permeability. It is noteworthy that the conventional medication cyclopamine has been demonstrated to permeate the blood-brain barrier.

Synthetic Accessibility (SA) reflects how easy it is to synthesize a compound, with ratings ranging from 1 (easily synthesizable) to 10 (difficult to synthesize). The SA value for Liriodenine was 2.77, compared to 2.61 for 2’,4-dihydroxy-3-methoxy chalcone. Cyclopamine, the standard drug, was identified as the most difficult molecule to synthesize with a SA value of 6.3.

Liriodenine has a Log S index value slightly higher than the benchmark of -4, and 2’,4-dihydroxy-3-methoxy chalcone demonstrates blood-brain barrier permeability. The molecular property violations do not impact a compound’s drug-like qualities unless they are proven to be toxic. The ADMET analysis suggests that Liriodenine and 2’,4-dihydroxy-3-methoxy chalcone possess the molecular characteristics required to be considered as potential drug candidates.

## 4. Discussion

In the current study, *insilico* screening using AutoDock and Gromacs was used to identify the compounds that have high affinity towards the four regulatory proteins of the Hedgehog pathway. The *insilico* studies are performed to understand the dynamics between two molecules virtually. These computational studies can be used to determine whether to invest our time and efforts in the *in vitro* analysis of the same. The objective of the current study was to determine the potential of the naturally occurring compounds to bind and interfere with the proteins of the Hedgehog pathway. The interaction of the compounds with the proteins was observed with docking analysis and its stability was realized in molecular dynamic simulations.

In the study the selected compounds are naturally occurring phytochemicals isolated from plants that are easily available. These compounds are well-known among phytochemicals with anticancer activity as they have been associated with various studies. Except for Cyclohexanone, 2,3,3-trimethyl-2-(3-methyl-1,3-butadienyl)-, (Z)- (chalcone derivative), Liriodenine (alkaloid) and 1-phthalanone (Ɣ-lactone) all are chalcones. Chalcones are one of the most bioactive molecules available in nature.

The study focuses on understanding the efficacy of these molecules in modulating the proteins of the Hedgehog pathway identified for their involvement in tumorigenesis of various cancers. These proteins include the Smoothened, Patched I, Gli II and Sonic Hedgehog proteins. *Insilico* studies to find the inhibitors to these proteins have been done in the past. Most of the studies emphasize only one protein, mostly the Smoothened. Docking and simulation studies against the Smoothened protein have tested many natural (alkaloids [70], berberine derivatives [71]) and synthetic compounds including established inhibitors [25, 72]. There are also *insilico* findings to Sonic Hedgehog [73, 74] and Gli II [24] proteins inhibitors with molecules such as Robotnikinin, traditional Chinese medicines and GANT61 respectively. The inhibitors are screened and binding is analyzed mostly against a single protein target.

The outcome of our study suggests two compounds, Liriodenine and 2’,4-dihydroxy-3-methoxy chalcone, as novel inhibitors to regulatory proteins of the Hedgehog pathway. Liriodenine engages three of the four regulatory proteins with greater binding affinity making it a suitable candidate to target multiple proteins. On the other hand, 2’,4-dihydroxy-3-methoxy chalcone exhibits higher binding affinity towards the Sonic Hedgehog protein. A closer look at the interaction of these compounds with the protein show that they share hydrogen-bonded interaction between them. The comparative analysis of docking shows that the Liriodenine has good binding affinity with Sonic Hedgehog protein as well, just falling short of 2’,4-dihydroxy-3-methoxy chalcone.

The molecular dynamic simulation analysis of the compound-protein complex infers the presence of stable interaction by forming hydrogen bonds throughout the 100 ns simulations. Both Liriodenine and 2’,4-dihydroxy-3-methoxy chalcone bind to their respective proteins with a minimum of three hydrogen bonds. This comes without any significant movement in the α-carbon molecules present in the backbone of the protein. This is important for the stability of the interaction necessary to complete the reaction.

The *insilico* pharmacokinetic and ADMET analysis result also concurs that Liriodenine and 2’,4-dihydroxy-3-methoxy chalcone can be used as drug candidates. Both the molecules show less toxicity high metabolism whereas they have good solubility and ease of synthesis. Especially Liriodenine, which show better pharmacokinetic characteristics than the standard drug cyclopamine.

## Supporting information

S1 Table Literature survey on the naturally occurring compounds. The table gives a summary of the various chalcones isolated from the commonly occurring plants. It also provides information on the cancerous cell lines that have been reportedly inhibited by the chalcones isolated from the respective extract.
(DOCX)

S2 Table Structures of the compounds used in analysis. The table has the 2D structures of the seventeen compounds used in the *insilico* analysis along with that of the standard cyclopamine.
(DOCX)

S1 Graphical abstract (TIF)
