# Supplementary material for: Computational investigation of naturally occurring anticancer agents in regulating Hedgehog pathway proteins
Source: PLoS One. 2024 Dec 3;19(12):e0311307. doi: 10.1371/journal.pone.0311307 (PMC11614240; doi:10.1371/journal.pone.0311307)
Supplement: S1 Table — The table gives a summary of the various chalcones isolated from the commonly occurring plants. It also provides information on the cancerous cell lines that have been reportedly inhibited by the chalcones isolated from the respective extract. (DOCX) [file pone.0311307.s001.docx]

# Supporting Information

Hedgehog pathway proteins modulation by naturally available anticancer agents: A computational study

Renu Pai^1¶^, Divijendranatha Reddy Sirigiri^1¶^, Rajyalakshmi Malempati^1^, Saisha Vinjamuri^1^*^¶^

^1^ Department of Biotechnology, BMS College of Engineering, Bengaluru, Karnataka, India.

*Email: saishav.bt@bmsce.ac.in (VS)

Phone: +91 9448093424

ORCID: 0000-0002-6136-5914

^¶^ These authors contributed equally to this work.

**Table S1** gives a summary of the various chalcones isolated from the commonly occurring plants. It also provides information on the cancerous cell lines that have been reportedly inhibited by the chalcones isolated from the respective extract.

| **Sl. No** | **Plant** | **Family** | **Chalcones isolated** | **Cell Lines** |
| --- | --- | --- | --- | --- |
| 1 | *Angelika keiskei* | Umbelliferae | Isobavachalcone Xanthoangelol  Xanthoangelol B  Xanthoangelol F  4’hydroxyderricin | IMR32[1]  Jurkat cells  HL60[2] |
| 2 | *Glycyrrhiza inflata* | Fabaceae | Isoliqiritigenin  Isoliquiritin  Licochalcone A  Licochalcone B | Ca Ski, SiHa,  HeLa, C-33A [3],  HeLa [4]  MG63[5] |
| 3 | *Mimosa tenuifolia* | Fabaceae | Kukulkanin A  Kukulkanin B | HCT15[6] |
| 4 | *Polyalthia cauliflora* | Annonaceae | 2’,4’ dihydroxy chalcone  2’,4’ dihydroxy, 3’ methoxy chalcone  Liriodenine | HL60  HeLa  MCF-7[7] |
| 5 | *Fissistigma launoginosum* | Annonaceae | Fissistin  Isofissistin  Pedicin | KB cells [8] |
| 6 | *Uvaria dulcis* | Annonaceae | 2’,3’ dihydroxy, 4’,6’ dimethoxy chalcone  Corresponding dihydrochalcone | HaCaT cells [9] |
| 7 | *Bridelia ferruginea benth* | Euphorbiaceae | 6’,4’ dihydroxy, 3’ propen chalcone | MCF7[10] |
| 8 | *Mallotus phillipensis* | Euphorbiaceae | Kamala chalcone A  Kamala chalcone B | THP-1[11] |
| 9 | *Sapium sebiferum* | Euphorbiaceae | Chalcone glycoside | HK-2[12] |

# References:

1. Tabata K, Motani K, Takayanagi N, Nishimura R, Asami S, Kimura Y, et al. Xanthoangelol, a Major Chalcone Constituent of Angelica keiskei, Induces Apoptosis in Neuroblastoma and Leukemia Cells. Biol Pharm Bull. 2005;28: 1404–1407. doi:10.1248/bpb.28.1404

2. Akihisa T, Kikuchi T, Nagai H, Ishii K, Tabata K, Suzuki T. 4-Hydroxyderricin from Angelica keiskei Roots Induces Caspase-dependent Apoptotic Cell Death in HL60 Human Leukemia Cells. J Oleo Sci. 2011;60: 71–77. doi:10.5650/jos.60.71

3. Hirchaud F, Hermetet F, Ablise M, Fauconnet S, Vuitton D, Prétet J-L, et al. Isoliquiritigenin Induces Caspase-Dependent Apoptosis via Downregulation of HPV16 E6 Expression in Cervical Cancer Ca Ski Cells. Planta Med. 2013;79. doi:10.1055/s-0033-1350956

4. Shibata S, Inoue H, Iwata S, Ma R, Yu L, Ueyama H, et al. Inhibitory Effects of Licochalcone A Isolated from Glycyrrhiza inflata Root on Inflammatory Ear Edema and Tumour Promotion in Mice. Planta Med. 1991;57: 221–224. doi:10.1055/s-2006-960078

5. Huang Z, Jin G. Licochalcone B Induced Apoptosis and Autophagy in Osteosarcoma Tumor Cells *via* the Inactivation of PI3K/AKT/mTOR Pathway. Biol Pharm Bull. 2022;45: 730–737. doi:10.1248/bpb.b21-00991

6. Kim H-G, Oh H-J, Ko J-H, Song HS, Lee Y-G, Kang SC, et al. Lanceoleins A–G, hydroxychalcones, from the flowers of Coreopsis lanceolata and their chemopreventive effects against human colon cancer cells. Bioorganic Chem. 2019;85: 274–281. doi:10.1016/j.bioorg.2019.01.003

7. Ghani NA, Ahmat N, Ismail NH, Alias A, Khairunissa N, Zawawi NA, et al. Flavonoid Analogues Isolated from the Stem Bark of Malaysian Polyalthia Cauliflora VAR. Cauliflora (Annonaceae) with Anticancer Properties.

8. Alias Y, Awang K, Hadi AHA, Thoison O, Sévenet T, Païs M. An Antimitotic and Cyctotoxic Chalcone from Fissistigma lanuginosum. In: ACS Publications [Internet]. American Chemical Society; 1 Jul 2004 [cited 31 Aug 2023]. doi:10.1021/np50122a002

9. Takada-Takatori Y, Tomii Y, Takemasa S, Takeda Y, Izumi Y, Akaike A, et al. Protective Effects of 2′,3′-Dihydroxy-4′,6′-dimethoxychalcone Derived from Green Perilla Leaves against UV Radiation-Induced Cell Injury in Human Cultured Keratinocytes. Biol Pharm Bull. 2019;42: 1936–1941. doi:10.1248/bpb.b19-00618

10. Vinjamuri S, Shanker D, Ramesh RS, Nagarajan S. IN VITRO EVALUATION OF HEMOLYTIC ACTIVITY AND CELL VIABILITY ASSAY OF HEXANOIC EXTRACTS OF BRIDELIA FERRUGINEA BENTH. World J Pharm Pharm Sci. 4.

11. Kulkarni RR, Tupe SG, Gample SP, Chandgude MG, Sarkar D, Deshpande MV, et al. Antifungal dimeric chalcone derivative kamalachalcone E from Mallotus philippinensis. Nat Prod Res. 2014;28: 245–250. doi:10.1080/14786419.2013.843178

12. Michl J, Bello O, Kite GC, Simmonds MSJ, Heinrich M. Medicinally Used Asarum Species: High-Resolution LC-MS Analysis of Aristolochic Acid Analogs and In vitro Toxicity Screening in HK-2 Cells. Front Pharmacol. 2017;8. Available: https://www.frontiersin.org/articles/10.3389/fphar.2017.00215
