# Supplementary material for: Computational investigation of naturally occurring anticancer agents in regulating Hedgehog pathway proteins
Source: PLoS One. 2024 Dec 3;19(12):e0311307. doi: 10.1371/journal.pone.0311307 (PMC11614240; doi:10.1371/journal.pone.0311307)
Supplement: S2 Table — The table has the 2D structures of the seventeen compounds used in the insilico analysis along with that of the standard cyclopamine. (DOCX) [file pone.0311307.s002.docx]

# Supporting Information

Hedgehog pathway proteins modulation by naturally available anticancer agents: A computational study

Renu Pai^1¶^, Divijendranatha Reddy Sirigiri^1¶^, Rajyalakshmi Malempati^1^, Saisha Vinjamuri^1^*^¶^

^1^ Department of Biotechnology, BMS College of Engineering, Bengaluru, Karnataka, India.

*Email: saishav.bt@bmsce.ac.in (VS)

Phone: +91 9448093424

ORCID: 0000-0002-6136-5914

^¶^ These authors contributed equally to this work.

**Table S2 gives the structures of all seventeen chalcones along the standard Cyclopamine**

| 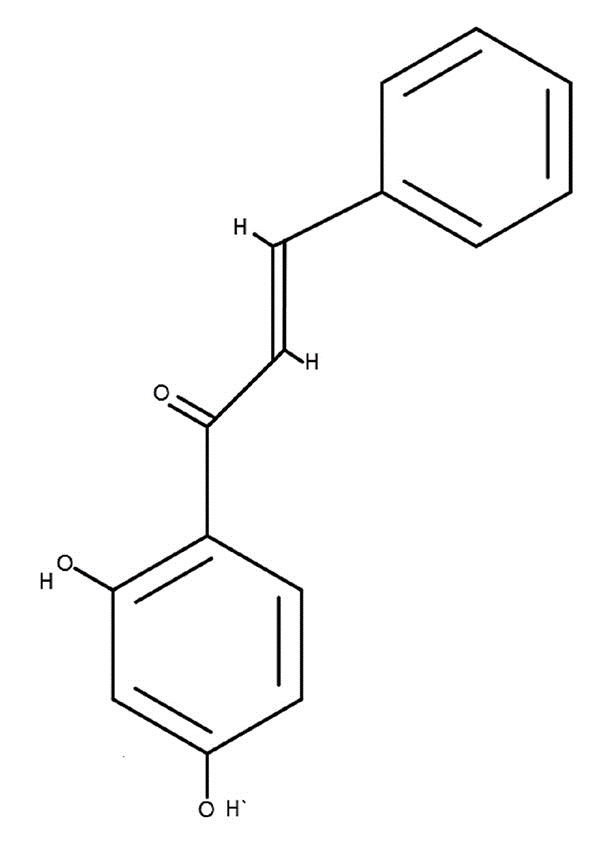 | 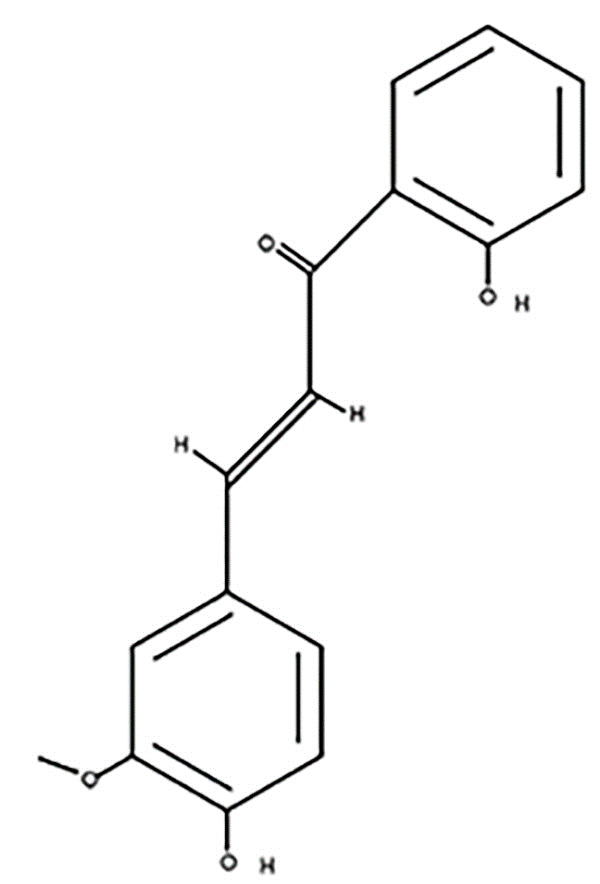 | 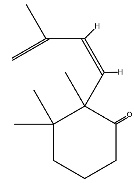 |
| --- | --- | --- |
| 2’, 4’-dihydroxy chalcone | 2’, 4’-dihydroxy, 3’-methoxy chalcone | Cyclohexanone-2,3,3-trimethyl-2-(3-methyl-1,3-butadienyl) |
| 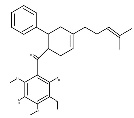 | 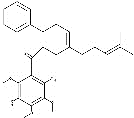 | 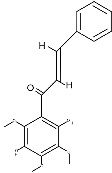 |
| Fissistin | Isofissistin | Pedicin |
| 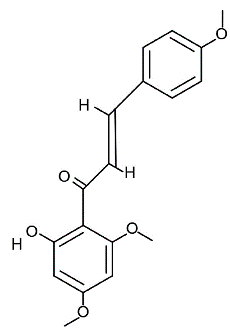 | 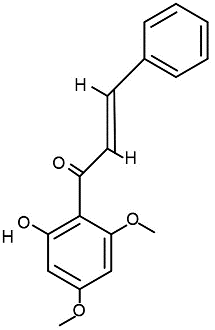 | 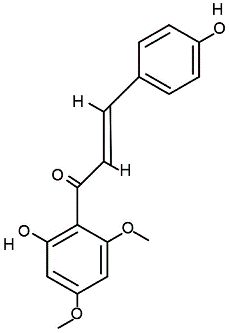 |
| Flavokawain A | Flavokawain B | Flavokawain C |
| 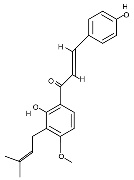 | 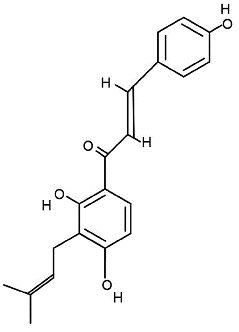 | 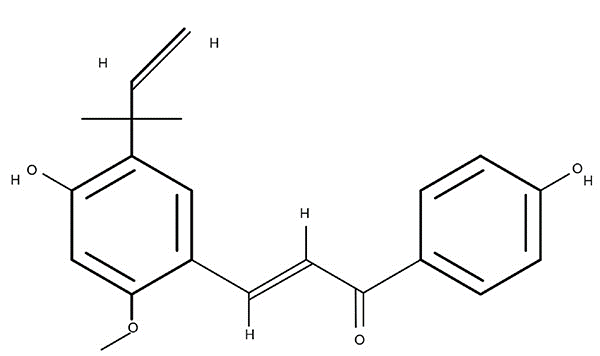 |
| Hydroxyderricin | Isobavachalcone | Licochalcone A |
| 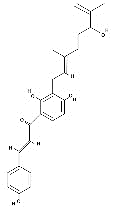 | 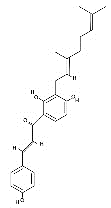 | 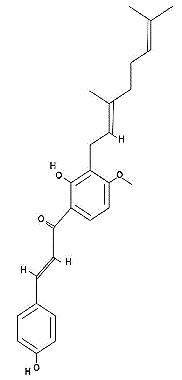 |
| Xanthoangelol B | Xanthoangelol | Xanthoangelol F |
| 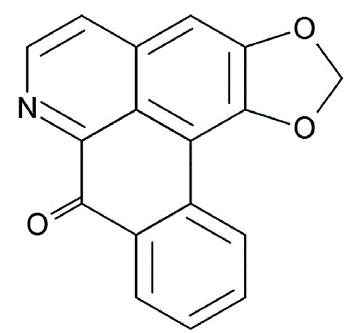 | 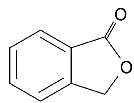 | 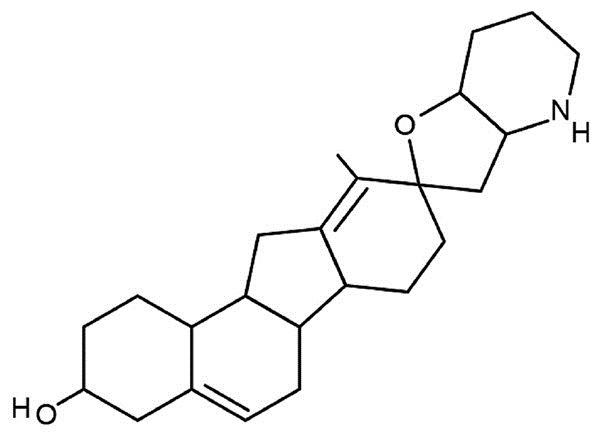 |
| Liriodenine | 1-Phthalanone | Cyclopamine |
