# Supplementary figures and images for: Computational investigation of naturally occurring anticancer agents in regulating Hedgehog pathway proteins
Source: PLoS One. 2024 Dec 3;19(12):e0311307. doi: 10.1371/journal.pone.0311307 (PMC11614240; doi:10.1371/journal.pone.0311307)

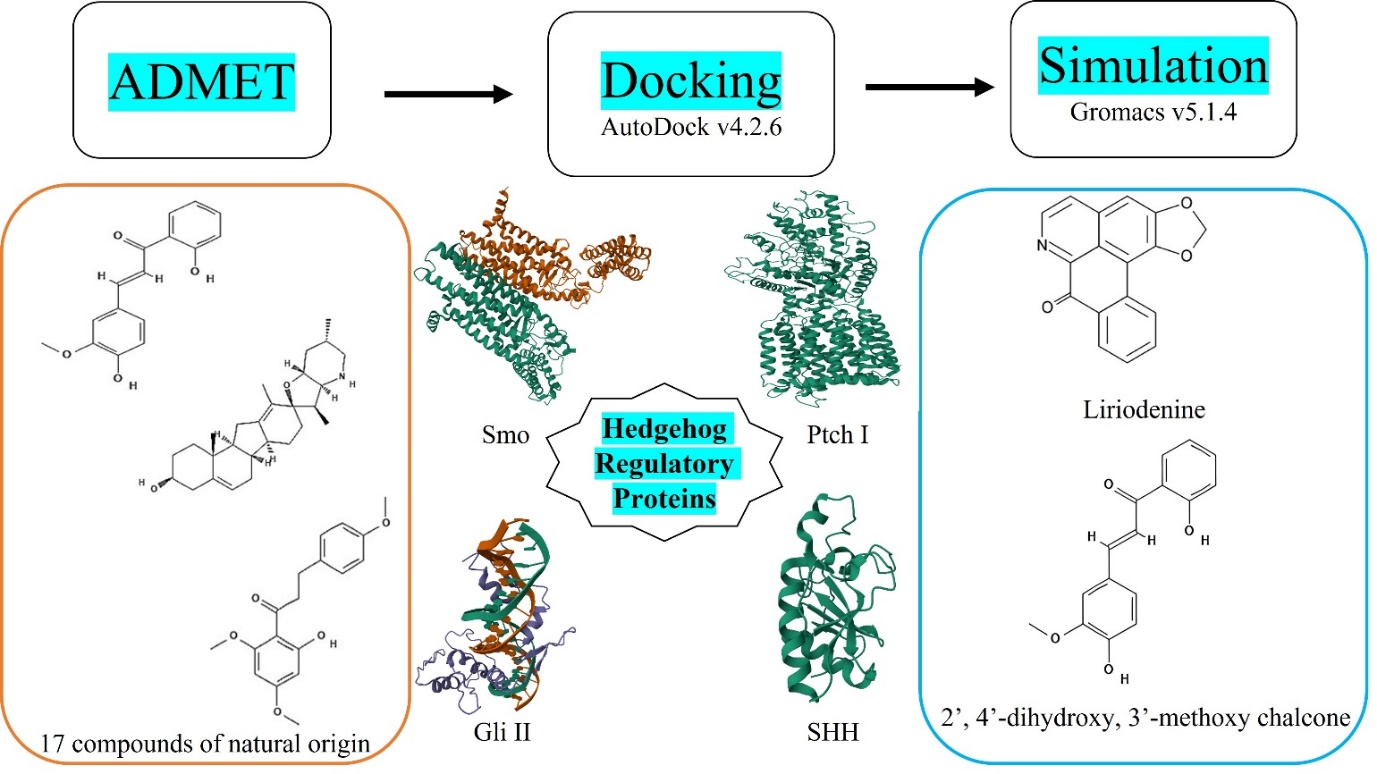

Supplement: S1 Graphical abstract — (TIF) [file pone.0311307.s003.tif]
